# Squamous Cell Carcinoma of the Tonsil Causing Small Bowel Perforation in the Setting of Metastasis and Treatment With Programmed Death-1 Inhibitors: A Case Report

**DOI:** 10.7759/cureus.10739

**Published:** 2020-09-30

**Authors:** Robert J Dabek, Anas Bizanti, Samantha Thomas, Abhishek Kalla, Isam Hamdallah

**Affiliations:** 1 Department of Surgery, Saint Agnes Hospital, Baltimore, USA; 2 Department of Internal Medicine, Saint Agnes Hospital, Baltimore, USA; 3 Department of Hematology and Oncology, Saint Agnes Hospital, Baltimore, USA

**Keywords:** head and neck neoplasms, gastrointestinal perforation, pd-1 inhibitors, visceral metastasis

## Abstract

Metastasis of extra-intestinal carcinoma to the gastrointestinal tract (GIT) is a rare event, most commonly occurring with malignant melanoma. Anti-PD-1 (programmed death-1) immunotherapeutic agents are immune checkpoint inhibitors with proven benefit across multiple cancer types, including squamous cell carcinoma of the head and neck (SCCHN). Here we describe a case of small bowel perforation attributed to a primary SCCHN metastasizing to the GIT in the setting of treatment with PD-1 inhibitors.

## Introduction

Extraintestinal metastasis to the small bowel is a rare event most commonly due to malignant melanoma [1]. Other cancers known to metastasize to the small bowel include, lung, breast, and renal neoplasms [2]. Metastasis to the gastrointestinal tract (GIT) from extraintestinal sources resulting in small bowel perforation is an even less common occurrence. A few prior case reports had reported lung adenocarcinoma and melanoma causing intestinal perforation [3-7]. There have been only a handful of reported cases of squamous cell carcinoma of the head and neck metastasizing to the small bowel and causing intestinal perforation, representing extremely rare events [8].

Anti-PD-1 (anti-programmed death-1) and anti-PD-L1 (anti-programmed death-ligand 1) agents are indicated for the treatment of melanoma, cutaneous squamous cell carcinoma, squamous cell carcinoma of the head and neck (SCCHN), non-small cell lung cancer (NSCLC), renal carcinoma, and Hodgkin’s lymphoma [9]. PD-1 and PD-L1 play a vital role in cell cycle checkpoint inhibition, providing downregulation of anti-tumor immunity. Thus, inhibition of this interaction via anti-PD-1 or anti-PD-L1 monoclonal antibodies is an effective therapy for tumor regression. The adverse effects of these novel therapeutic agents are not yet fully understood. The term immune-mediated adverse reactions (IMARs) was created to describe these adverse effects of these drugs, which most frequently included fatigue, skin disorders, gastrointestinal issues, and endocrine dysfunction. Rare events such as pneumonitis, hepatitis, encephalitis, renal injury, colitis, hypercalcemia, and pleural effusion have also been reported [9].

Here we describe a case of small bowel perforation attributed to a primary SCCHN metastasizing to the GIT in the setting of treatment with PD-1 inhibitors. This is the first reported case of a palatine tonsillar cancer metastasizing to the small bowel, and adds to the small number of reported bowel perforations resulting from treatment with monoclonal PD-1 and PD-L1 inhibitors in the presence of metastatic tumor from any primary source [10-13].

## Case presentation

A 56-year-old man with a history of alcohol abuse and liver cirrhosis was recently diagnosed with nonresectable T1N2bM0 stage IVa p16-squamous cell carcinoma of the right palatine tonsil. He completed treatment with cisplatin and radiotherapy eight months ago at which time CT and PET scan showed a decrease in tumor size and activity; however several positive hilar lymph nodes were identified. Treatment was continued with pembrolizumab (Keytruda®) every three weeks. During the course of his treatment, a subjective reduction of tumor size on physical exam was noted. The patient complained of fatigue, difficulty sleeping, constipation, and muscle cramps during the treatment course. He subsequently developed a left-sided Bell’s palsy. Laboratory testing revealed a pancytopenia with a reduction in all cell lines (WBC 2.8, RBC 2.95, platelet count 98 k/uL). He was briefly placed on a course of steroids which did not resolve Bell's palsy, and due to metastatic nodal disease pembrolizumab was resumed.

**Figure 1:**
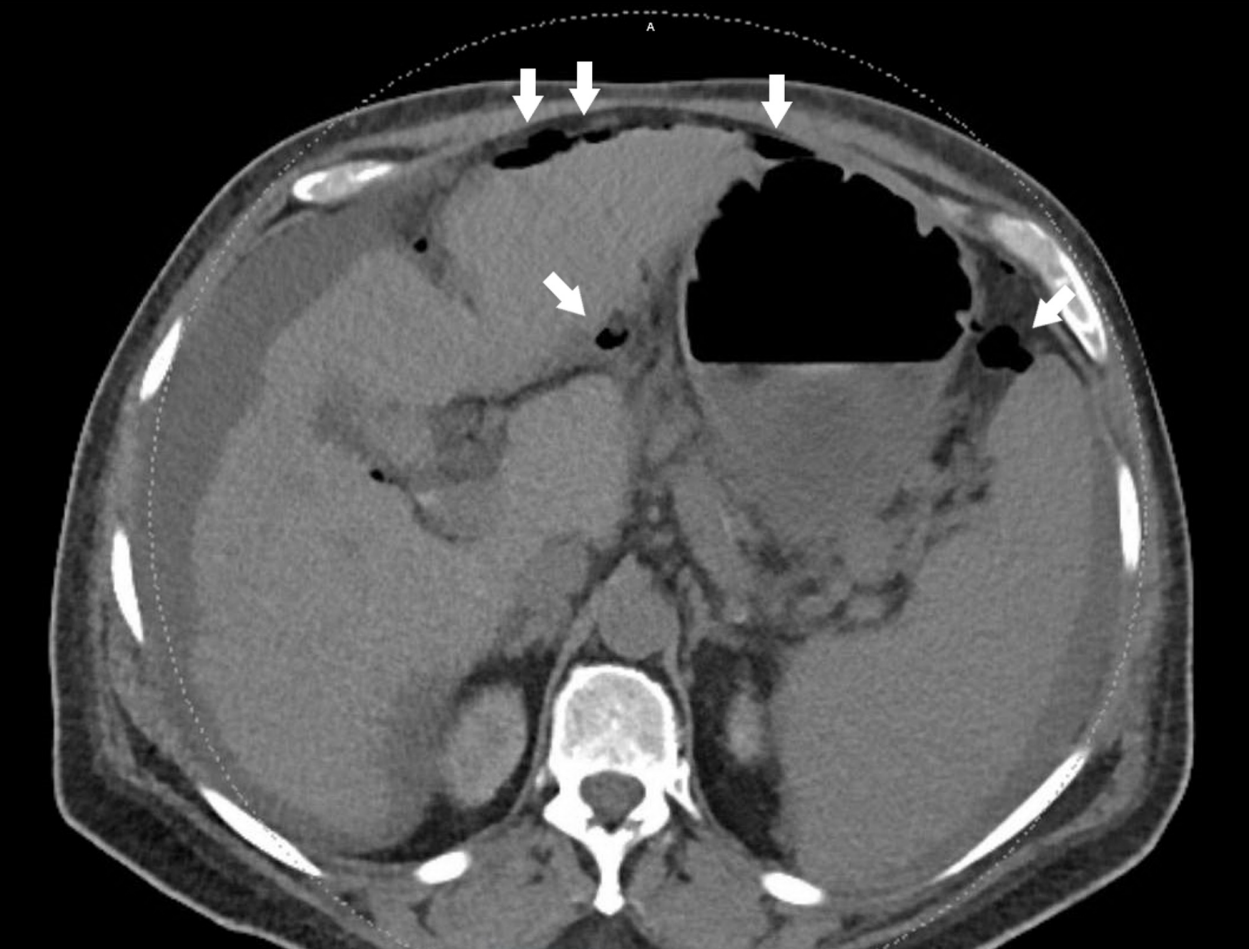
Axial noncontrast CT of abdomen demonstrating free air (arrows) around the stomach and anterior abdominal wall

Approximately two weeks after his last dose of immunotherapy, he presented to the emergency department with a complaint of fever and abdominal pain. He denied nausea, vomiting, diarrhea, or constipation prior to arrival. On presentation, he was febrile (100.5 F) and tachycardic (136 bpm) with physical exam findings suggestive of peritonitis with a rigid, distended, and diffusely tender abdomen. A noncontrast CT of the abdomen and pelvis was performed in the emergency room prior to surgical consultation, which demonstrated pneumoperitoneum and mild diffuse small bowel wall thickening (Figure 1).

**Figure 2:**
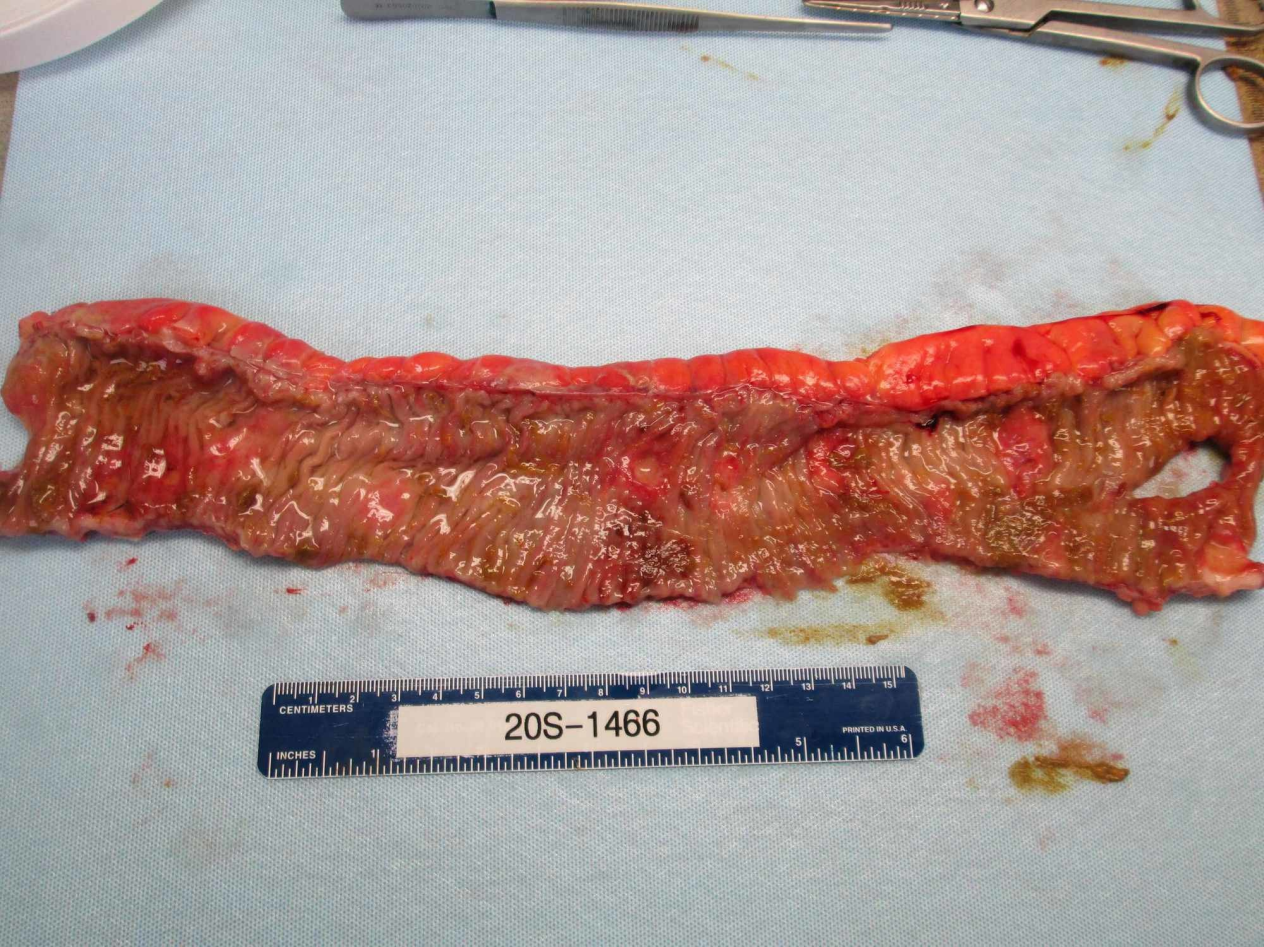
Gross photograph of segment of small bowel showing grossly evident perforation, measuring approximately 25 mm in greatest dimension

**Figure 3:**
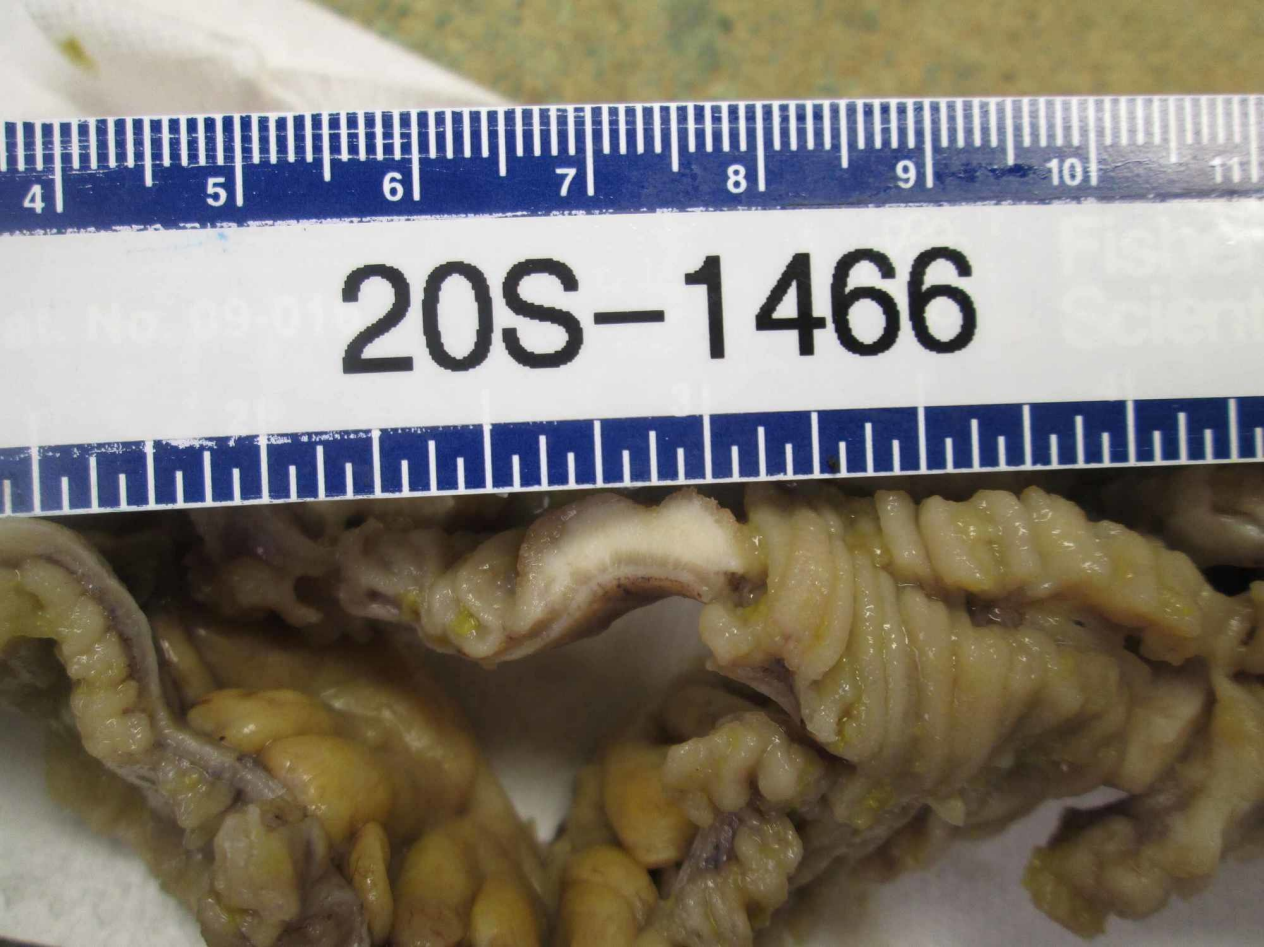
Gross photograph showing an intramural nodule, measuring approximately 10 mm in length and 4 mm in thickness

**Figure 4:**
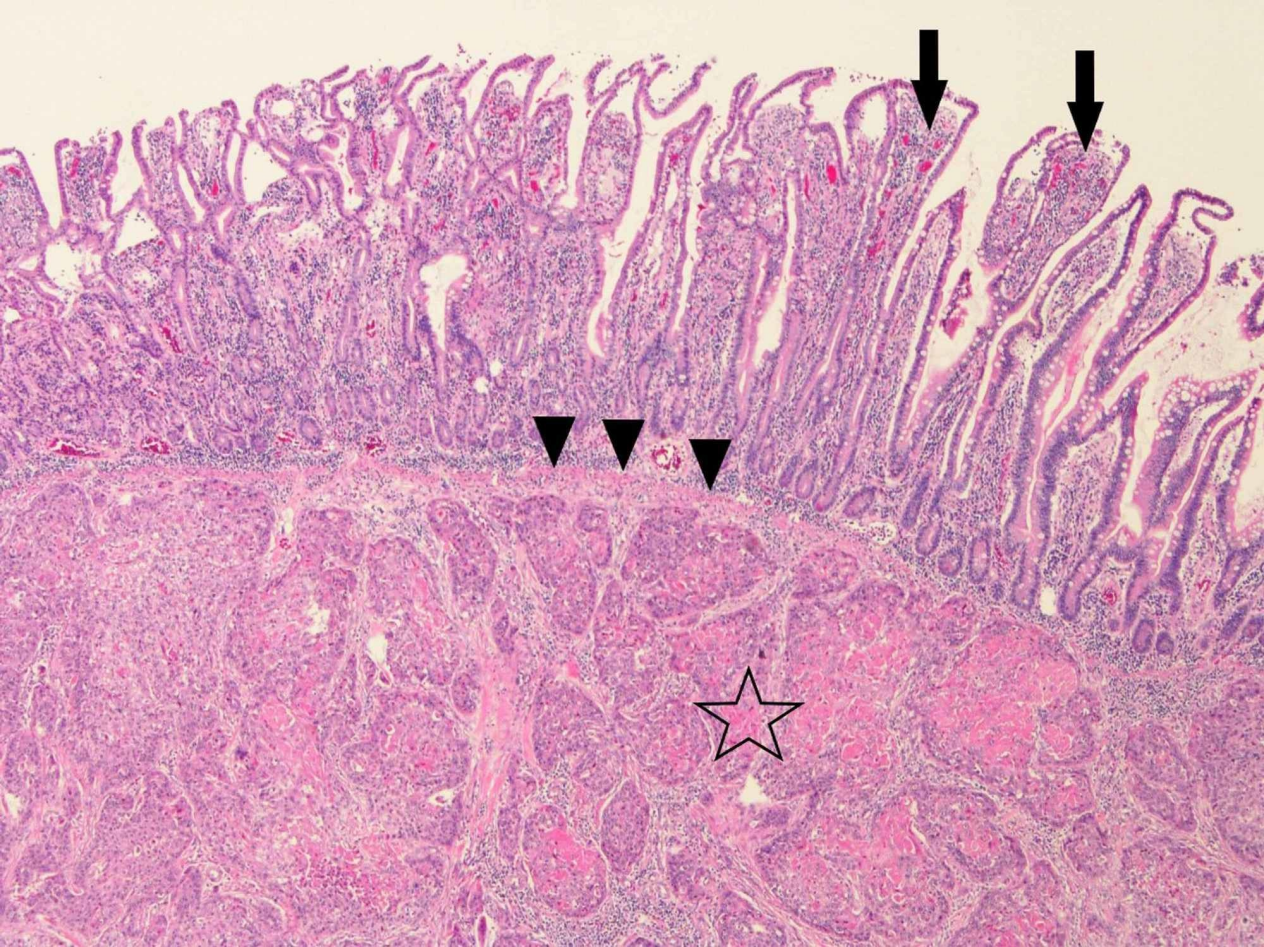
Low power view showing intestinal villi (arrows) and a nodule of metastatic squamous cell carcinoma and inflammatory infiltrate (star) lying between the mucosa and the muscularis propria (arrowheads) of the small bowel (H&E stain, 80X magnification) H&E, hematoxylin and eosin

He underwent an emergent exploratory laparotomy. Approximately 3 liters of ascitic fluid was evacuated from the peritoneal cavity. Examination of the bowel revealed numerous plaque-like lesions of the bowel wall with involvement of the entire length of the small intestine from the ligament of Treitz to the ileocecal valve. One lesion involving the ileum was found to be perforated. An approximately 20 cm segment of ileum containing this perforated lesion was resected and an end to end anastomosis was performed using a linear stapler (Figure 2). The abdomen was closed, and the patient was admitted to the intensive care unit due to septic shock requiring vasopressor support. He recovered post-operatively without any immediate complications. Pathological examination of the specimen revealed multiple small well-circumscribed firm lesions (Figure 3). Histologically evident squamous cell carcinoma with some inflammatory infiltrate was found at multiple foci, consistent with metastatic SCCHN (Figure 4). Upon further discussions and counseling with medical oncology, the patient decided to proceed with hospice care.

## Discussion

Cancer of the tonsil is treated with by a surgical, oncologic, or combined approach. The rates of tonsillar cancer have been increasing with the increasing prevalence of HPV infection [14]. The most common site of metastasis for SCCHN, including tonsil SCC, are lung, liver, and bone [15]. We were unable to identify any prior reports of a palatine tonsil SCC causing small intestinal perforation as the first metastatic sign. As such, this is the first report, and the perforation may be attributed to the use of pembrolizumab, a PD-1 inhibitor. However, other causes of perforation including evolution of metastatic disease can not be ruled out. Intestinal perforation is not a known risk of PD-1 inhibitor therapy. However, gastrointestinal symptoms are not uncommonly seen in patients treated with PD-1 inhibitors. Diarrhea, constipation, abdominal pain and colitis have been listed as adverse effects of pembrolizumab, as outlined in the FDA package insert [16]. The cause of intestinal perforation in patients treated with PD-1 inhibitors may be due to direct destruction of tumor cells, resulting in areas of necrosis and weakness. Alternatively, pseudoprogression may be the cause of perforation. Pseudoprogression refers to the resulting enlargement of tumor due to local infiltration of inflammatory cells [17]. This is caused by the inflammatory response to tumor cell death after treatment with anti-cancer therapies. In the described case, the pathology samples showed a nodule of metastatic squamous cell carcinoma laying between the mucosa and the muscularis propria with inflammatory cell infiltration, secondary to the use of Pembrolizumab, favoring the pseudoprogression mechanism (Figure 4). The patient did not exhibit any major gastrointestinal symptoms prior to this presentation except for chronic constipation, which was symptomatically controlled with stool softeners. He presented with sudden abdominal pain which was later revealed to be due to intestinal perforation. Although pseudoprogression is not commonly seen in SCCHN, the use of Pembrolizumab can facilitate this inflammatory process [17]. It’s imperative for clinicians to keep a broad suspicion of the possibility of metastasis (known and unknown) resulting in gastrointestinal perforation while initiating PD-1 inhibitor therapy.

## Conclusions

There have been a small number of case reports of metastatic lung cancer and melanoma causing small bowel perforation in the setting of PD-1 inhibitor use. The mechanism of perforation is likely caused by immunomodulator-mediated destruction of tumor cells, or as a consequence of local infiltration of lymphocytes. Finally, this is the first reported case of metastasis of SCCHN to the small bowel causing a perforation, which may be attributed to immunotherapy with a PD-1 inhibitor. Although this is an extremely rare event, clinicians should be aware of this possibility.
